# Impact of Zika Virus Infection on Human Neural Stem Cell MicroRNA Signatures

**DOI:** 10.3390/v12111219

**Published:** 2020-10-27

**Authors:** Denna Tabari, Catharina Scholl, Michael Steffens, Sandra Weickhardt, Fabian Elgner, Daniela Bender, Marie-Luise Herrlein, Catarina Sabino, Vesselina Semkova, Michael Peitz, Andreas Till, Oliver Brüstle, Eberhard Hildt, Julia Stingl

**Affiliations:** 1 Research Division, Federal Institute for Drugs and Medical Devices, 53175 Bonn, Germany; Denna.Tabari@bfarm.de (D.T.); Michael.Steffens@bfarm-research.de (M.S.); Sandra.Weickhardt@bfarm.de (S.W.); 2 Department of Virology, Paul-Ehrlich-Institut, 63225 Langen, Germany; Fabian.Elgner@icloud.com (F.E.); Daniela.Bender@pei.de (D.B.); Marie-Luise.Herrlein@pei.de (M.-L.H.); Catarina.Sabino@pei.de (C.S.); Eberhard.Hildt@pei.de (E.H.); 3 Institute of Reconstructive Neurobiology, LIFE & BRAIN Center, University of Bonn Medical Faculty & University Hospital Bonn, 53127 Bonn, Germany; vsemkova@lifeandbrain.com (V.S.); peitz@uni-bonn.de (M.P.); a.till@uni-bonn.de (A.T.); r.neuro@uni-bonn.de (O.B.); 4 Cell Programming Core Facility, Medical Faculty, University of Bonn, 53172 Bonn, Germany; 5 Department of Clinical Pharmacology, University Hospital, RWTH Aachen University, 52074 Aachen, Germany; jstingl@ukaachen.de

**Keywords:** Zika virus, microRNA (miRNA), pathogenesis, extracellular vesicles, neural stem cells, flavivirus

## Abstract

Zika virus (ZIKV) is a mosquito-borne virus, which can cause brain abnormalities in newborns, including microcephaly. MicroRNAs (miRNAs) are small non-coding RNAs, which post- transcriptionally regulate gene expression. They are involved in various processes including neurological development and host responses to viral infection, but their potential role in ZIKV pathogenesis remains poorly understood. MiRNAs can be incorporated into extracellular vesicles (EVs) and mediate cell-to-cell communication. While it is well known that in viral infections EVs carrying miRNAs can play a crucial role in disease pathogenesis, ZIKV effects on EV-delivered miRNAs and their contribution to ZIKV pathogenesis have not been elucidated. In the present study, we profiled intracellular and EV-derived miRNAs by next generation sequencing and analyzed the host mRNA transcriptome of neural stem cells during infection with ZIKV Uganda and French Polynesia strains. We identified numerous miRNAs, including miR-4792, which were dysregulated at the intracellular level and had altered levels in EVs during ZIKV infection. Integrated analyses of differentially expressed genes and miRNAs showed that ZIKV infection had an impact on processes associated with neurodevelopment and oxidative stress. Our results provide insights into the roles of intracellular and EV-associated host miRNAs in ZIKV pathogenesis.

## 1. Introduction

Zika virus (ZIKV), a mosquito-borne virus and member of the Flaviviridae family, was initially isolated in 1947 from a rhesus monkey in the Zika forest in Uganda [1]. In 2007 the first human epidemic was recorded on Yap Island, followed by a major outbreak in French Polynesia in 2013 [2,3]. In the course of this outbreak, ZIKV infection was for the first time linked to Guillain-Barré Syndrome and severe neurodevelopmental defects, including microcephaly [4,5,6,7]. International awareness of the potential for severe disease outcomes grew in 2015 when ZIKV emerged in Brazil and subsequently spread to most of the Americas in 2016 [8,9,10]. During the global spread, the virus diversified and presently ZIKV strains are classified into African and Asian/American lineages. ZIKV has circulated for decades in Africa without any documented clinical relevance. It is unclear whether genetic change might have resulted in a greater capacity of neurovirulence or whether ZIKV has always been teratogenic and severe cases have not been documented before 2013 [11,12]. Due to the rapid spread of the virus and its severe effects on infants, on 1st February 2016, the World Health Organization (WHO) declared a Public Health Emergency of International Concern (PHEIC) [13]. Since then, significant progress has been made in elucidating the effects of ZIKV on the host transcriptome; however, the molecular mechanisms by which ZIKV leads to microcephaly are still not deeply understood. Previous studies have shown that ZIKV can infect human neural stem cells (hNSCs) and human neural progenitor cells (hNPCs), thus disrupting pathways involved in differentiation, apoptosis and cell cycle control [14,15,16,17].

MicroRNAs (miRNAs) are a class of small non-coding RNA molecules (containing about 22 nucleotides) that act as post-transcriptional regulators of mRNA. By binding to partially complementary sites of the target mRNA, miRNAs can suppress the translation [18]. MiRNAs play an important role in the regulation of genes involved in various processes including development, differentiation and apoptosis [19,20,21]. MiRNAs can further be incorporated into extracellular vesicles (EVs) and serve as signaling molecules to mediate intercellular communication [22]. Various Flaviviridae viruses modulate intracellular and EV-associated miRNA profiles, which can have an impact on the pathogenesis [23,24]. In the context of ZIKV infection, the relevance of EV-derived miRNAs has remained unexplored. Since only a few studies have analyzed the effects of ZIKV infection on global miRNA regulation at the intracellular level, their role in ZIKV pathogenesis is not fully understood [25,26]. Kozak et al. have found that ZIKV infection alters the host transcriptomic profiles in astrocytes and dysregulates miRNAs involved in the unfolded protein response pathway, which is a cellular response mechanism to endoplasmic reticulum (ER) stress [26].

Accumulating evidences have linked ER stress and oxidative stress to the pathogenesis of Flaviviridae viruses, including Dengue virus (DENV) and hepatitis C virus (HCV) [27]. An essential factor in the regulation of oxidative stress is the transcription factor Nrf2 (nuclear factor erythroid 2 (NF-E2)-related factor 2), which triggers the expression of numerous cytoprotective genes [28,29]. Under unstressed conditions, Nrf2 is associated with the actin binding protein Keap1 (Kelch-like ECH associating protein 1) and localized within the cytoplasm. Keap1 targets Nrf2 for rapid proteasomal degradation though ubiquitination, thereby maintaining it at relatively low levels in the cell [30]. Upon oxidative stress, Nrf2 dissociates from Keap1 and translocates into the nucleus, where it forms a heterodimer with small Maf (sMaf) proteins and subsequently binds to the antioxidant response element (ARE). Nrf2/ARE-regulated genes play a crucial role in cellular adaptation to reactive oxygen species (ROS) and some viruses manipulate the antioxidant system for their own benefit [28,31,32]. Little is currently known about the role of oxidative stress and the underlying molecular details during ZIKV infection in neuronal tissue on neurodevelopmental effects [33].

In this study, we investigated the role of miRNA-mediated gene expression during ZIKV infection of induced pluripotent stem cell (iPSC)-derived hNSCs. To gain more knowledge on the mechanisms of pathogenesis of neurodevelopmental damage, we profiled host miRNA and mRNA transcriptomes and performed integrative analyses.

## 2. Materials and Methods

### 2.1. Cell Culture

Long-term self-renewing neuroepithelial-like stem cells (lt-NES^®^) represent a reductionist in vitro model for the early stages of human neural stem cell differentiation [34]. These cells, which are derived from both human embryonal stem cells (hESCs) [34] and induced pluripotent stem cells (iPSCs) [35], retain a constant neuro- and gliogenic potential even after long-term proliferation. Lt-NES^®^ cells used in the present study were generated from iPSC line iLB-C-31f [36], which is derived from a female donor in her mid-20s by retroviral reprogramming. Routine characterization of the resulting cell line was performed as depicted in the supplemental information (Figures S1 and S2). The cells were maintained on plastic 6-well cell culture dishes coated with 1x poly-L-ornithine and 10 mg/mL laminin (Merck, Darmstadt, Germany) in Dulbecco’s modified Eagle’s/F12 medium (DMEM-F12) (Thermo Fisher Scientific, Waltham, MA, USA) supplemented with N2 Supplement (PAA Laboratories, Pasching, Austria) and containing 10 ng/mL basic fibroblast growth factor 2 (FGF2), 10 ng/mL epidermal growth factor (EGF) (both from R&D Systems, Minneapolis, MN, USA) and 1:1000 B-27 Supplement (Thermo Fisher Scientific, Waltham, MA, USA). Passaging was performed every 2–3 days by splitting at a 1:2 ratio. In short, cells were detached using incubation with 0.125% trypsin (Thermo Fisher Scientific, MA, USA) for 5–10 min at room temperature. Trypsin was deactivated with trypsin inhibitor (Thermo Fisher Scientific, MA, USA) and the cells were spun down at 300× *g* for 3 min. Supernatant was aspirated and the cell pellet was resuspended in maintenance medium and plated onto poly-L-ornithine/laminin pre-coated cell culture dishes.

### 2.2. Infection

Two ZIKV strains were used in the study: ZIKV Uganda 976 (kindly provided by the European Virus Archive), for which no associations with severe symptoms have been documented, and ZIKV PF13/251013-18 from French Polynesia (kindly provided by Professor Musso from the Institute Louis Marladé in Tahiti and by the European Virus Archive), which has been linked to severe pathological effects [2]. The lt-NES^®^ cells were seeded into 6-well plates (1 million cells per well) and infected with a multiplicity of infection (MOI) of 0.1 or 1 using serum free virus stocks. MOI of 1 was chosen with the aim of seeing more significant effects. The medium of the infected and uninfected cells was changed daily (24, 48 and 72 h post infection) and the supernatants were collected 8 h after medium change for further analyses by plaque assay. Serum free virus stocks were generated by infection of Vero cells with the respective virus strain in Dulbecco’s Modified Eagle’s Medium (DMEM) high glucose (BioWest, Nuaillé, France) supplemented with 10% fetal bovine serum superior (Biochrom GmbH, Berlin, Germany), 2 mM L-glutamine (Biochrom GmbH, Berlin, Germany), 100 U/mL penicillin and 100 μg/mL streptomycin (Paul-Ehrlich-Institut facilities, Langen, Germany). On day 3 post infection, cells were washed once with phosphate-buffered saline (PBS) followed by a medium change to DMEM supplemented with 10% KnockOut^TM^ serum replacement (Thermo Fisher Scientific, Waltham, MA, USA). On day 7 post infection the supernatant was harvested, centrifuged for 5 min at 1000× *g* and stored at −80 °C until determination of titer by plaque assay and infection of lt-NES^®^ cells.

### 2.3. Plaque-Forming-Assay

Plaque assay was performed to quantify infectious viral particles. Vero cells were seeded in 6-well plates (3 × 10^5^ per well) and infected with 100 µl of serial dilutions from cell culture supernatants. After 2 h, the medium was removed and the cells were layered with 0.4% agarose solution (SeaPlaque^®^ Agarose; Lonza, Basel, Switzerland). Five days after infection, the agarose overlay was removed and cells were fixed with 4% formaldehyde in PBS for 20 min. Visualization of the plaques was achieved by staining with crystal violet (0.1% in 20% ethanol; Merck, Darmstadt, Germany) 15 min at room temperature. After washing with distilled water to remove excess crystal violet solution, the plates were dried, and plaques were counted.

### 2.4. MiRNAs: Sequencing and Data Analysis

For the analysis of intracellular miRNAs, total RNA was extracted from infected and uninfected lt-NES^®^ cells at 24, 48 and 72 hpi using miRNeasy Mini Kit (217004, Qiagen, Hilden, Germany) according to the manufacturer’s instructions. For the analysis of EV-derived miRNAs, cell culture supernatants were collected at the indicated time points and EV-associated miRNAs were extracted using exoRNeasy Serum/Plasma Kit (77064, Qiagen, Hilden, Germany) as described by the manufacturer. RNA sequencing libraries were generated using the NEBNext Mulitplex Small RNA Library Prep Set for Illumina (E7300S, New England Biolabs, Frankfurt, Germany) following size selection by running the samples on a 10% polyacrylamide gel and isolating the 147 bp. Samples were purified using the QIAquick PCR Purification Kit (28104, Qiagen, Hilden, Germany). Libraries were validated on a high sensitivity DNA chip using the 2100 Bioanalyzer (5067-4626, Agilent, Santa Clara, CA, USA). Next generation sequencing was conducted on the Illumina^®^ platform using MiSeq Reagent Kit v2 (50 cycles) (MS-102-2001, Illumina, CA, USA). To analyze the miRNA sequencing data, fastq files were generated and adapter trimming was performed with the program cutadapt. Reads were aligned to the whole human genome using bowtie2. The package DESeq2 was used to calculate the fold change, *p*-value (Wald test) and adjusted *p*-value (Benjamini Hochberg Adjustment) of differentially expressed miRNAs. To focus on miRNAs that potentially have a greater impact on host transcripts, miRNAs with copy numbers lower than 100 were removed from further data analysis [37]. Gene ontology (GO) analyses of biological processes and pathways of KEGG (Kyoto Encyclopedia of Genes and Genomes), which is a knowledge database for systematic analysis of gene functions, were performed using DIANA mirpath v3 [38,39]. Putative targets were determined using the homology search algorithm microT-CDS and TarBase, which is a database of experimentally validated miRNA-gene interactions (*p*-value threshold of 0.05) [40]. For the microT-CDS prediction, a microT threshold of 0.8 was set. The miRNA sequencing data sets have been deposited in Gene Expression Omnibus (GEO) database (GSE157532).

### 2.5. Microarray-Based Gene Expression Analysis

Gene expression profiling was performed using SurePrint G3 Human Gene Expression 8x60K Microarray Kit (Agilent One Color Microarray Technology, Agilent Technologies, Santa Clara, CA, USA). Of the total RNA, 100 ng was extracted using miRNeasy Mini Kit (217004, Qiagen, Hilden, Germany) according to the manufacturer’s instructions and was used for amplification.

Microarray results were extracted using Agilent Feature Extraction version 11.0.1.1 and analyzed using Genespring 14.9.1 GX software (Agilent Technologies, Santa Clara, CA, USA). To reduce artefacts, signal intensities below 50 were removed from the dataset. A two-way ANOVA test was used as statistical analysis and *p*-values were adjusted for multiple test correction using the Benjamini Hochberg algorithm. Genespring 14.9.1 GX software was further used for fold change analyses and to crosscheck the list of differentially expressed genes with putative miRNA targets obtained from bioinformatics databases (PiCTar, TarBase, TargetScan, miRDB) [41,42,43,44]. Pathways and network patterns associated with significantly dysregulated genes were analyzed using Reactome version 73 (https://reactome.org) [45]. The microarray data sets have been deposited in GEO (GSE157532).

### 2.6. Real-Time Quantitative PCR

Real-time quantitative PCR (qRT-PCR) was performed to validate selected genes and miRNAs. According to the manufacturer’s instructions, cDNA was generated from 500 ng of total RNA for the validation of miRNAs using miScript II RT Kit (218161, Qiagen, Hilden, Germany) and from 1000 ng of total RNA for the validation of mRNA using QuantiTect Rev. Transcription Kit (205311, Qiagen, Hilden, Germany). Quantitative PCR was performed with the SYBR Green PCR Kit (204145, Qiagen, Hilden, Germany) using a Roche LightCycler 480 (Roche, Basel, Switzerland). For miRNA expression analyses, miScript Primer Assays were used (Hs_miR-205_1; Hs-miR-182_2; Hs_miR-4792-1; Hs-miR-16_2, Qiagen, Hilden, Germany) and Ct values were normalized to miR-16 levels. Gene expression was analyzed using Quantitect Primer Assays (Hs_ASNS_1_SG; Hs_FOXC1_2_SG; Hs_SESN2_1_SG; Hs_GAPDGH_1_SG, Qiagen, Hilden, Germany) and Ct values were normalized to GAPDH. Fold changes were calculated with the ∆∆Ct method [46].

### 2.7. Oxyblot

Carbonylation of proteins by ROS was analyzed using the OxyBlot^TM^ protein oxidation detection kit (Merck, Darmstadt, Germany) according to the manufacturer’s instructions. Briefly, ZIKV-infected cells and uninfected cells were harvested after 24 h, 48 h and 72 h, washed with PBS and lysed with RIPA buffer. For each sample the same amount of protein (15 µg) was used. Band intensity values were obtained using Image Studio Lite Software Version 5.2 (LI-COR Biosciences, Lincoln, NE, USA).

### 2.8. Immunofluorescence Microscopy

The cells were seeded on cover slides and fixed with 4% formaldehyde in PBS for 20 min at room temperature. Permeabilization and blocking were performed as described by Elgner et al. [47]. The cells were incubated with anti-ZIKV NS1 (1:1000; Biofront Technologies, Tallahassee, FL, USA) and anti-MafG (1.300; Abcam, Cambridge, UK) and afterwards with the secondary antibodies anti-mouse IgG-Alexa 488 (1:1000; Thermo Fisher Scientific, MA, USA) and anti-rabbit-Cy3 (1:400; Jackson Immunoresearch, West Grove, PA, USA). Nuclei were stained using 4′,6-diamidino-2-phenylindole (DAPI) (Carl Roth, Karlsruhe, Germany). Finally, the stained coverslips were mounted with Mowiol (Merck, Darmstadt, Germany) and analyzed using the confocal laser scanning microscope LSM 510 Meta and ZEN 2009 software (Carl Zeiss, Oberkochen, Germany).

### 2.9. Statistical Analysis

Statistically significant differences between group means were assessed by 2-tailed, unpaired Student’s t test and visualized using GraphPad Prism 8 software. Differentially expressed miRNAs from sequencing data were analyzed using DESeq2. A two-way ANOVA test was used as a statistic test for the microarray gene expression data. An adjusted *p*-value ≤ 0.05 was considered statistically significant.

## 3. Results

### 3.1. ZIKV Uganda and French Polynesia Alter Gene Expression in lt-NES^®^ Cells

**Figure 1:**
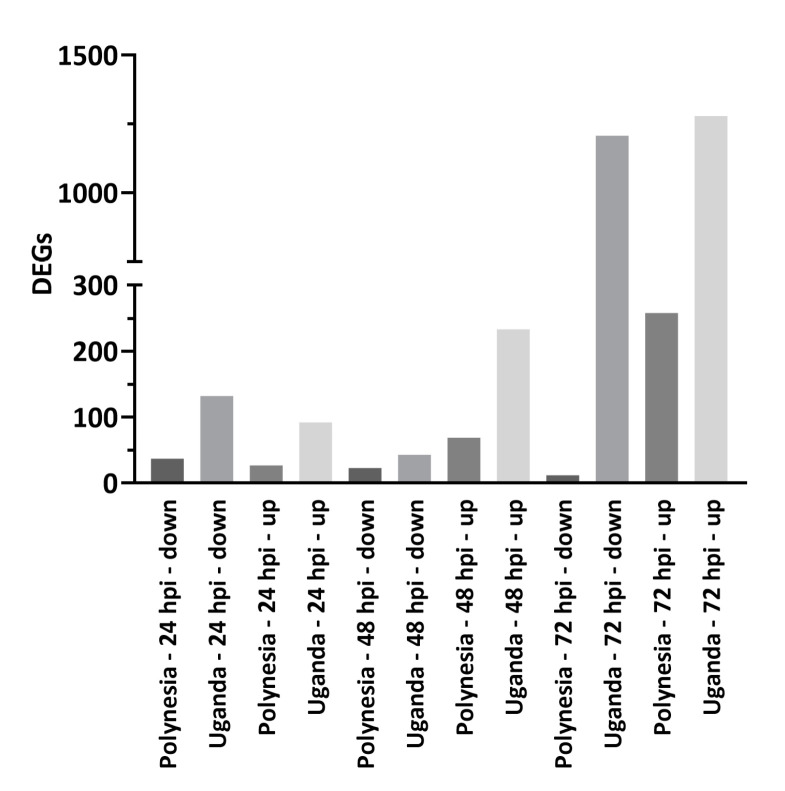
Differentially expressed genes (DEGs). Number of ≥ two-fold that were significant (*p*-value ≤ 0.05) and differentially expressed genes at 24, 48 and 72 h post infection with ZIKV Polynesia and ZIKV Uganda.

**Figure 2:**
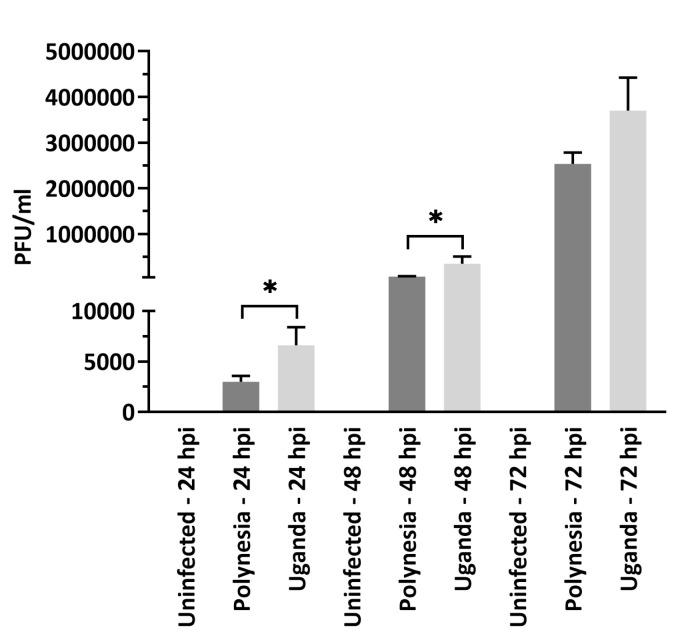
Plaque forming assays. Quantification was performed in Vero cells by plaque forming assays and the number of infectious viral particles is expressed in plaque forming units per mL (PFU/mL). Error bar represents the mean ± SEM of *n* = 3 biological replicates; * *p* < 0.05. Quantification of the number of extracellular infectious viral particles from lt-NES^®^ cells infected with either the Polynesia or the Uganda strain at 24, 48 and 72 hpi at multiplicity of infection (MOI) of 0.1.

To investigate the impact of ZIKV infection on gene expression and host miRNA regulation in a well characterized model for human neural stem cells, we analyzed the mRNA transcriptome of infected lt-NES^®^ cells at 24, 48 and 72 hpi. To this aim, lt-NES^®^ cells were infected using a MOI of 0.1 with either the French Polynesia PF13/251013-18 (ZIKV Polynesia) or the Uganda 976 strain. The Uganda strain in general had a higher impact on gene expression in lt-NES^®^ cells (Figure 1). By more than two-fold, ZIKV Uganda significantly upregulated 1280 genes and downregulated 1208 genes at 72 hpi, whereas only 258 genes were upregulated and 12 genes were downregulated by ZIKV Polynesia at the same time point. Plaque-forming assays revealed a significantly higher number of infectious particles produced by Uganda than by Polynesia strains at 24 and 48 hpi and no significant difference at 72 hpi (Figure 2).

**Figure 3:**
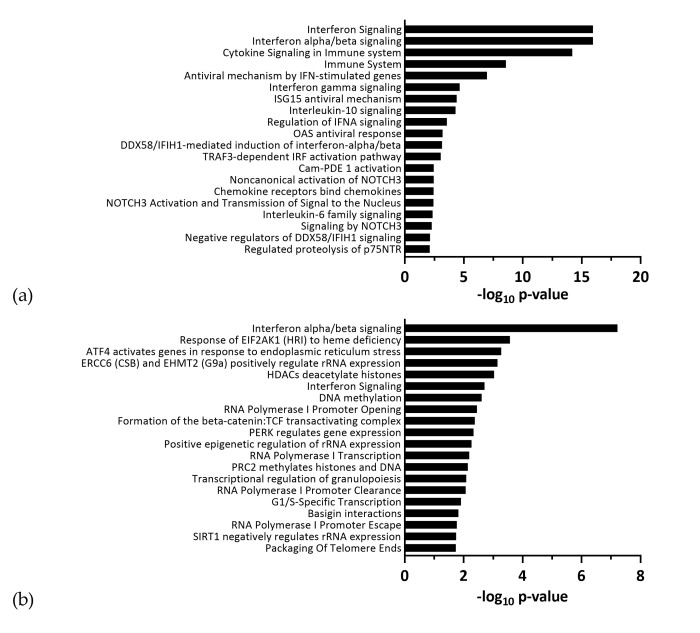
Top 20 enriched pathways identified by functional reactome analysis. Pathway analysis of ≥ two-fold up- and downregulated genes upon infection with either (**a**) ZIKV Polynesia or (**b**) ZIKV Uganda. Pathways ranked according to −log_10_ *p*-value.

**Figure 4:**
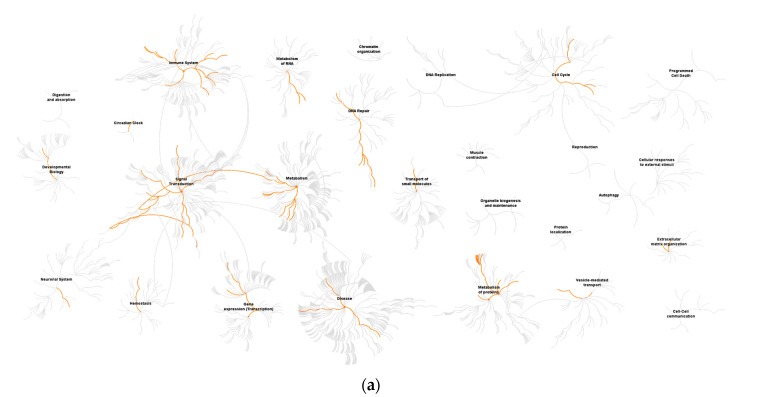

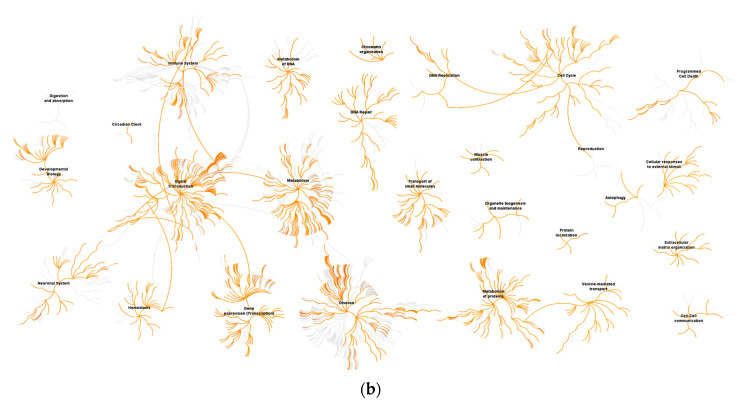
Reactome pathway diagram showing enriched signaling pathways as red lines. Genome-wide overview pathway analysis of ≥ two-fold downregulated genes for (**a**) ZIKV Polynesia and (**b**) ZIKV Uganda. The red lines denote over-representation of those pathways.

To identify in which processes the differentially expressed genes were involved, we performed pathway analyses using the reactome pathway database. The top ranked terms for ZIKV Uganda and Polynesia primarily included antiviral response related pathways (Figure 3). Among the most affected processes by ZIKV Uganda, cell cycle related pathways including G1/S-specific transcription were also significantly enriched. Both strains induced upregulation of genes, which are mainly involved in processes related to antiviral interferon signaling, ER stress and the unfolded protein response pathway, which is consistent with previous reports [25,26]. Downregulated genes were largely involved in signal transduction, cell cycle and gene expression related pathways (Figure 4, Tables S1 and S2). Using the comparative toxicogenomics database [48], which provides information about gene–disease relationships, we screened all differentially expressed genes for association with microcephaly. Although for the Uganda strain no neuropathological symptoms are documented, the screening revealed five genes related to microcephaly, which were only dysregulated by ZIKV Uganda and one gene, namely *ASNS* (asparagine synthetase), which was upregulated by both strains (Table 1). RT-qPCR analyses showed a higher upregulation of *ASNS* by ZIKV Uganda than by the Polynesia strain (Figure S3).

Overall, our results indicate an increased expression of immune response related genes over time (Table 2 and Table 3) including genes that are known to act as restriction factors for other viruses, namely *BST2* (tetherin, Figure S4) and *IFIT2* (interferon-induced protein with tetratricopeptide repeats 2) [49,50].

Taken together, our results show that ZIKV Uganda and Polynesia modulate the gene expression pattern in lt-NES^®^ cells by causing a dysregulation of genes mainly involved in interferon signaling and cell cycle, which may contribute to ZIKV pathogenesis.

### 3.2. Dysregulated miRNAs Play a Role in Neurodevelopment and Cell Cycle

**Figure 5:**
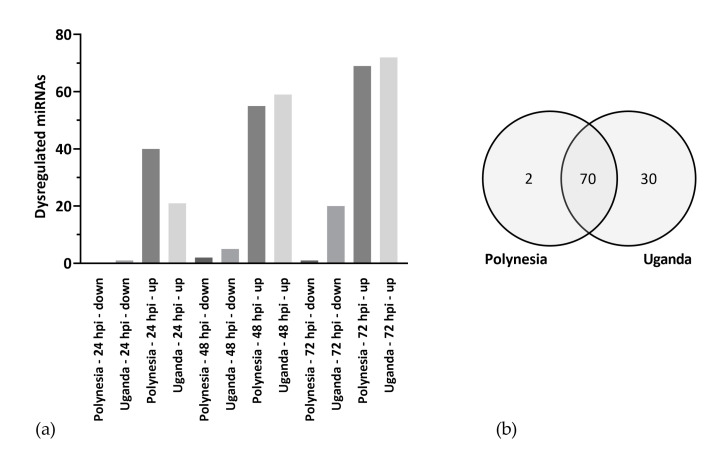
Dysregulated miRNAs. (**a**) Number of more than two-fold, significantly (*p*-value ≤ 0.05) dysregulated miRNAs by ZIKV Polynesia and Uganda at 24, 48 and 72 hpi. (**b**) Significantly more than two-fold dysregulated miRNAs by ZIKV Polynesia and Uganda including all time points.

**Figure 6:**
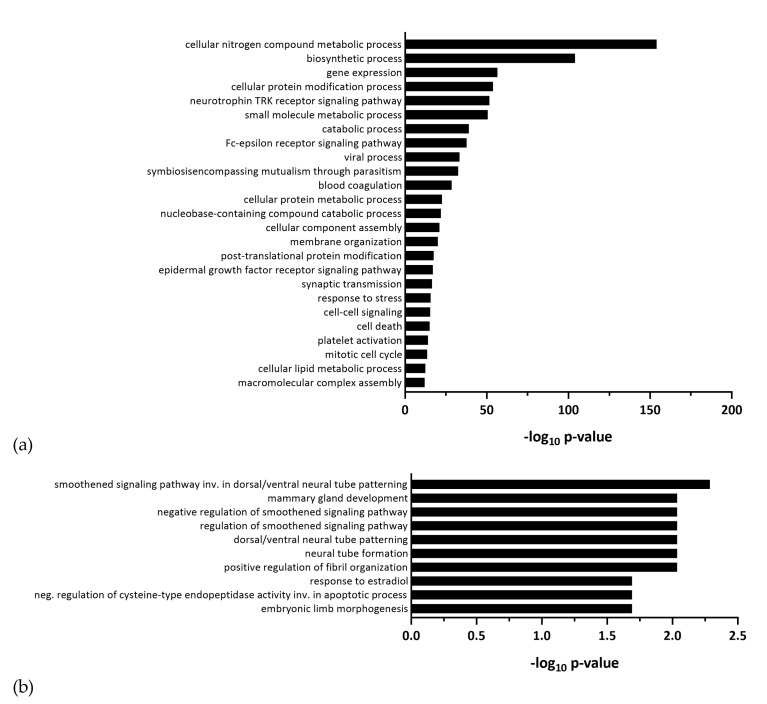
Gene ontology (GO) analysis of potential target genes of differentially expressed miRNAs. (**a**) GO analysis of biological processes of putative targets of miRNAs which were dysregulated in lt-NES^®^ cells during infection with ZIKV Polynesia and Uganda. (**b**) GO analysis of biological processes of putative targets of miR-7704, which was upregulated by ZIKV Polynesia and Uganda.

Since miRNAs regulate posttranscriptional gene expression and can play an important role in pathological processes during viral infections, we profiled differential miRNA regulation upon ZIKV infection. Similar to our gene expression analysis, we analyzed miRNA profiles of lt-NES^®^ cells infected with ZIKV Uganda and Polynesia at 24, 48 and 72 hpi at MOI of 0.1. Both virus strains induced dysregulation of miRNAs, whereby overall more miRNAs were upregulated than downregulated (Figure 5a). Interestingly, we found a large overlap between the ZIKV strains, both dysregulating the same 70 miRNAs (Figure 5b, Table S3), with 69 miRNAs upregulated and only one miRNA downregulated by both strains, namely miR-182-5p. To further characterize the differentially regulated miRNAs, potential target genes were predicted using the microT-CDS (v 5.0) database and gene ontology analysis was performed with a focus on biological processes. MiRNAs dysregulated by both strains were involved in the neurotrophin TRK receptor signaling pathway, synaptic transmission, and mitotic cell cycle, among others (Figure 6a). These processes were also enriched for the total of 30 miRNAs, which were dysregulated only by ZIKV Uganda (Table S4, Figure S9).

One specific miRNA—miR-7704—which was upregulated by ZIKV Polynesia and by ZIKV Uganda, showed up with potential roles in developmental processes including neural tube formation (Figure 6b). To further identify miRNA–mRNA interactions during ZIKV infection in lt-NES^®^ cells, we crosschecked the list of potential miRNA targets with mRNAs that were differentially expressed after ZIKV infection of lt-NES^®^ cells. This integrative analysis revealed various genes downregulated by ZIKV Uganda with roles in neurodevelopment, such as *EPHB4* (Ephrin Receptor B4) and *CNP* (2′,3′-Cyclic Nucleotide 3′ Phosphodiesterase),which may be depressed by numerous miRNAs that were upregulated in lt-NES^®^ cells. Thus, multiple upregulated miRNAs could potentially affect the same mRNA targets. Two miRNAs were significantly downregulated only by ZIKV Polynesia, namely miR-433-5p and miR-205-5p (Figure S5). Interestingly, miR-205-5p potentially targets the upregulated gene *ASNS* (Asparagine Synthetase) [51]. However, further experiments should be performed to assess the interaction between miR-205-5p and *ASNS* in lt-NES^®^ cells.

Collectively, our findings indicate an alteration of miRNA regulation following ZIKV infection in lt-NES^®^ cells, which was broadly similar between ZIKV Uganda and ZIKV Polynesia. We detected dysregulated miRNAs that might play a role in neurodevelopment and cell cycle related pathways and could be involved in ZIKV pathogenesis.

### 3.3. ZIKV Modifies the Host miRNA Cargo in Secreted EVs

**Figure 7:**
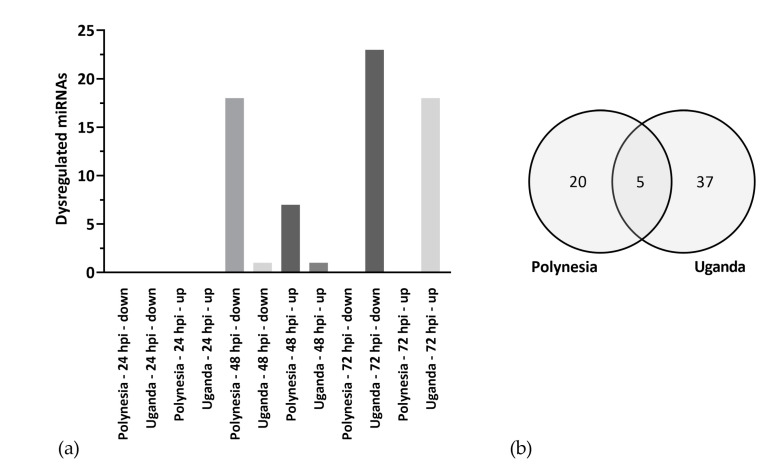
Level of extracellular vesicles (EV)-derived miRNAs. (**a**) Significantly (*p*-value ≤ 0.05) ≥ two-fold dysregulated mature miRNAs, which were incorporated into EVs during infection of lt-NES^®^ cells with either ZIKV Polynesia or Uganda. (**b**) EV-derived miRNAs with levels significantly (*p*-value ≤ 0.05) altered (≥two-fold) following infection with ZIKV Polynesia and Uganda at any time point.

**Figure 8:**
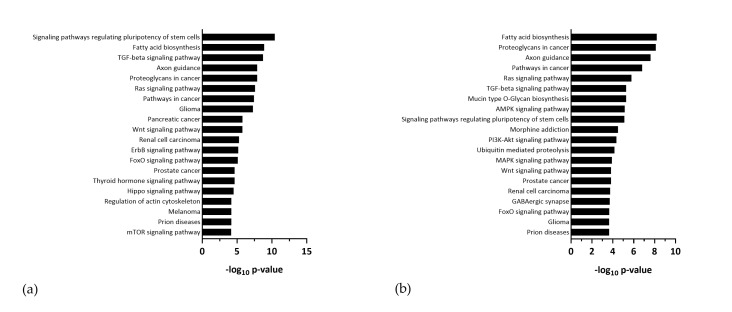
KEGG Pathway analysis of potential targets of EV-derived miRNAs which were differentially incorporated following infection with (**a**) ZIKV Polynesia and (**b**) ZIKV Uganda.

Since exosome-derived miRNAs are crucial for cell–cell communication and can be a central player in disease pathogenesis during viral infection, our next aim was to study how ZIKV infection affects the level of host miRNAs incorporated into EVs. To this aim, we analyzed EV-derived miRNAs 24, 48 and 72 h post infection of lt-NES^®^ cells at MOI of 1. Uninfected cells kept under the same conditions were used as a control. We found altered levels of EV-associated miRNAs by ZIKV Uganda, increasing over time. In contrast, for ZIKV Polynesia the effect on the signature of EV-derived miRNAs was higher at 48 hpi (Figure 7a). We detected five miRNAs within EVs with levels altered by both virus strains (Figure 7b). Using Diana mirPath v3 we performed KEGG pathway analyses of potential targets for EV-associated miRNAs with altered levels upon infection (listed in Tables S5 and S6), which revealed an enrichment of signaling pathways involved in neurodevelopmental processes including Wnt signaling, Pi3K-Akt signaling and signaling pathways regulating pluripotency of stem cell, among others (Figure 8). Both strains modulated the level of EV-associated miRNAs, which were involved in FoxO signaling and axon guidance. Interestingly, EV-derived miR-4792 was the only miRNA for which we found both an increased level following infection with ZIKV Polynesia (by 1.4-fold) and ZIKV Uganda (by 2.9-fold) at 48 hpi and additionally an upregulation by both strains at the intracellular level at any time point (Figure S6), which we validated by RT-qPCR. Using Diana mirPath v3 we further performed a gene ontology analysis of potential miR-4792 targets predicted by Tarbase, which uses experimentally validated data. Potential miR-4792 targets were involved in processes including in utero embryonic development and negative regulation of oxidoreductase activity, respectively (Table 4). Because of previously described interactions between miR-4792 and *FOXC1* (Forkhead Box C1) in various cell lines [20,52] we analyzed the expression of *FOXC1* mRNA in lt-NES^®^ cells during ZIKV infection. RT-qPCR analyses revealed that *FOXC1* was slightly downregulated by both ZIKV strains (Figure S7). However, further studies should be performed to investigate if *FOXC1* is repressed due to the upregulation of miR-4792 in lt-NES^®^ cells upon ZIKV infection.

Taken together, we observed that ZIKV infection induces multiple changes in secretion of host miRNAs in EVs. Overall, these EV-associated miRNAs, particularly miR-4792, were involved in oxidative and neurodevelopmental processes, suggesting that they could be a central player in ZIKV-induced neuropathogenesis given the important role of EVs in intercellular communication.

### 3.4. Dysregulated miRNAs and mRNAs Involved in Oxidative Stress

**Figure 9:**
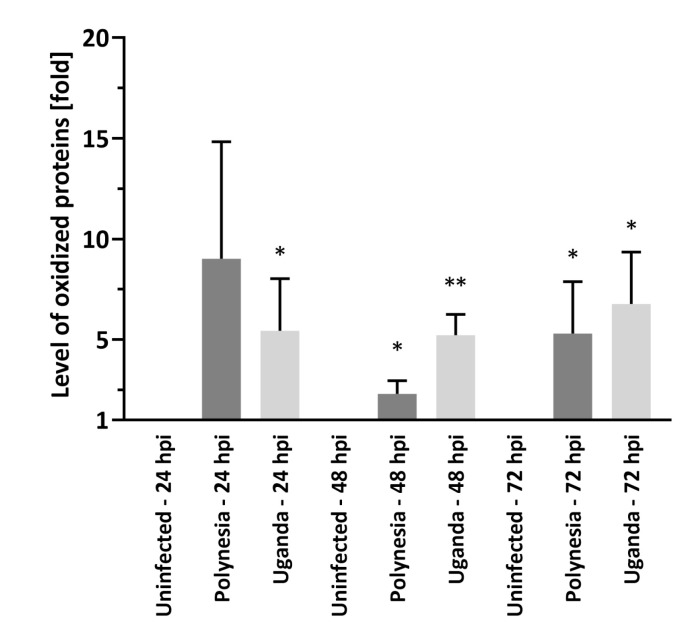
ZIKV infection induces elevated levels of oxidized proteins. Oxyblot analysis of lysates derived from uninfected lt-NES^®^ cells and lt-NES^®^ cells, which were infected with ZIKV Uganda and ZIKV Polynesia at MOI of 1 at 24, 48 and 72 hpi. Protein oxidation was analyzed by incubation with 2,4,-dinitrophenylhydrazine (DNPH) for covalent modification of oxidative modified proteins. Error bar represents the mean ± SEM of *n* = 3 biological replicates; * *p* < 0.05; ** *p* < 0.01.

**Figure 10:**
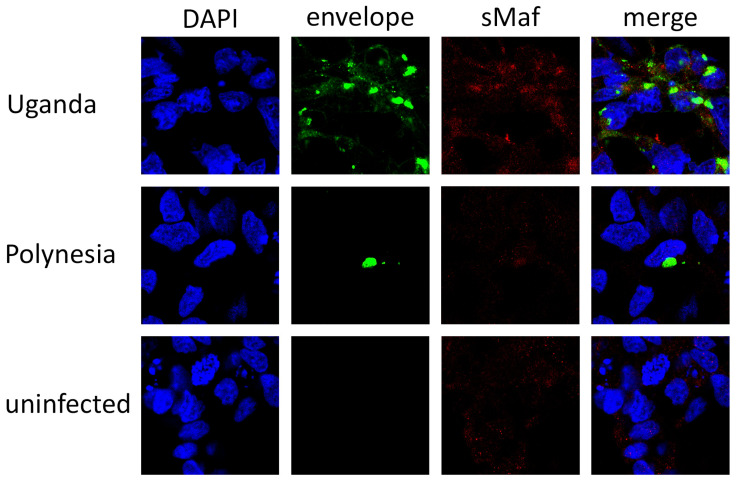
Increased amount of sMaf proteins in ZIKV infected cells. Confocal immunofluorescence microscopy of ZIKV-positive cells (ZIKV Uganda at 48 hpi and ZIKV Polynesia at 24 hpi) or negative control cells (24 hpi). The immunofluorescence staining was performed using the polyclonal sMaf-specific serum and an envelope specific antibody. Nuclei were stained with DAPI.

Since previous studies have linked ER stress and ROS increase to the pathogenesis of several Flaviviridae virus infections [33], we investigated how ZIKV infection affects miRNA-dependent regulation of genes related to oxidative stress and ER stress. To first assess if oxidative stress is induced by ZIKV infection, the carbonylation of proteins by ROS was quantified using oxyblots. Thereby the level of oxidative modified proteins was significantly higher after infection with either ZIKV Uganda or Polynesia compared with uninfected cells at 48 and 72 hpi, indicating an induction of oxidative stress during ZIKV infection (Figure 9 and Figure S10). We next studied the effect of ZIKV on Nrf2/ARE signaling, an antioxidant pathway known to be hijacked by other viruses [31]. To this aim, we analyzed the influence of ZIKV infection on the amount and subcellular distribution of sMaf proteins by immunofluorescence analysis. We could observe a colocalization of ZIKV Uganda and Polynesia envelope proteins with sMaf, as well as an enrichment of sMaf proteins in perinuclear regions after infection (Figure 10). In addition, RT-qPCR analysis showed a significant upregulation of the antioxidant gene Sestrin 2 (Figure S8a). Interestingly, miR-182-5p was downregulated upon ZIKV infection which potentially targets the gene Sestrin2, as described by Lin et al. [53] (Figure S8b).

We next performed a gene ontology analysis to generate a list of differentially expressed genes upon infection with ZIKV Polynesia, which were significantly annotated to the terms response to oxidative stress and response to endoplasmic reticulum stress. Our analysis revealed 19 upregulated genes, which are listed in Table 5 and Table 6, including *DDIT3* (DNA-damage-inducible transcript 3), *XBP1* (X-box binding protein 1) and *EIF2AK2* (eukaryotic translation initiation factor 2-alpha kinase 2), among others. To identify if these genes could be targeted by miRNAs, which were downregulated during infection with ZIKV Polynesia, we performed an integrative analysis using Tarbase. Our analysis revealed that miR-205-5p, which was downregulated at the intracellular level, could potentially target the gene *ATF3* (activating transcription factor 3), besides *ASNS*. Furthermore, we found multiple EV-associated miRNAs with decreased levels upon infection with ZIKV Polynesia, which potentially regulate the same mRNA targets implicated in oxidative stress or ER stress (Table 7).

Taken together, we observed that during ZIKV infection, oxidative stress is induced, and we identified a number of potential miRNA-mRNA interactions related to oxidative stress and ER stress, which were differentially regulated and may contribute to ZIKV pathogenesis.

## 4. Discussion

ZIKV infection induces severe neurodevelopmental damage with microcephaly observed in children of women infected during pregnancy. However, the pathogenesis of these viral induced effects on neurogenesis are not yet fully understood. In this study, we used a human neural stem cell (NSC) model system and integrative methods to assess whether infection of NSCs with ZIKV may affect miRNA signatures and gene expression profiles. We analyzed intracellular host miRNAs and measured changes in levels of miRNAs incorporated into EVs in response to ZIKV infection. We identified a variety of signaling pathways that were altered during infection and may contribute to the development of brain malformations.

Despite the important role of miRNAs in the pathogenesis of flavivirus infections [54,55], only a few studies have been conducted to analyze how ZIKV could alter global miRNA regulation, and their potential involvement in ZIKV pathogenesis and microcephaly has not yet been fully elucidated [25,26,56]. Through global miRNA transcriptome analyses, we found many overlaps in the intracellular miRNA profiles modulated by the ZIKV strains Uganda and French Polynesia. However, ZIKV Uganda had a greater impact on mRNA and miRNA dysregulation, which might be a result of its higher infection level [25].

Both virus strains caused a dysregulation of numerous pathways involved in neurodevelopment and cell cycle, suggesting that they may contribute to the microcephaly phenotype. Interestingly, we found several differentially expressed genes following infection with ZIKV Uganda, including *COL4A1*, *MIR17HG*, *TUBA1A*, *SLC2A1* and *STAMBP*, which are associated with microcephaly according to the comparative toxicogenomics database [48]. Accumulating evidence reveals that African strains of ZIKV can cause equal or worse damage to neuronal cells compared to Asian lineages [11,57]. Our findings support the hypothesis that ZIKV Uganda could also influence neurodevelopment and that clinical signs have remained undetected due to insufficient capacity to detect rare cases in Africa before 2013 [12,58]. However, another hypothesis is that differences between Asian and African ZIKV lineages regarding cytopathic effects might influence pathogenicity and that Asian ZIKV may cause chronic infections within the central nervous system due to its lower ability to cause cell death [59,60,61].

We further identified the gene *ASNS* to be differentially expressed by both strains, which has previously been associated with developmental disorders including microcephaly [62,63]. *ASNS* encodes the protein asparagine synthetase, a metabolic enzyme that converts aspartate and glutamine to asparagine and glutamate in an ATP-dependent reaction [64]. A dysregulation of the balance of amino acids in the brain induced by ZIKV could be a potential contributing factor to the microcephaly phenotype [62,63]. The overexpression of *ASNS* following ZIKV infection could be related to the downregulation of miR-205-5p, which has previously been described to target *ASNS* [51], although further experiments should be performed to study this interaction in lt-NES^®^ cells.

Recent studies have highlighted the role of EV-associated miRNAs in the pathogenesis of several viruses, however, changes in EV-derived miRNA levels during ZIKV infection have not yet been described [65,66]. Our findings revealed that ZIKV infection of lt-NES^®^ cells altered the signature of EV-associated miRNAs. Differentially incorporated miRNAs in EVs produced in response to ZIKV infection were associated with processes related to neural and embryonic development, including Axon guidance and FoxO signaling.

We found miR-4792 to be highly upregulated by both ZIKV strains at the intracellular level and significantly enriched in EVs secreted from ZIKV infected cells. Previous studies by other groups have described that overexpression of miR-4792 can suppress the growth of nasopharyngeal tumors in mice and induce cell cycle arrest and activate apoptosis in A459 cells [20,52]. It is possible that overexpression of miR-4792 following ZIKV infection could induce cell death of lt-NES^®^ cells and through transmission via EVs this could even affect uninfected neighboring cells and thus be a possible mechanism for impaired development of the brain. Furthermore, miR-4792 has been described to directly target the transcription factor FOXC1 in both, nasopharyngeal carcinoma cells and A549 cells [20,52]. According to previous studies, FOXC1 is required for normal cerebellar development and impairment of FOXC1 function contributes to the development of Dandy–Walker syndrome [67,68], which is a brain malformation associated with ZIKV infection [69]. Since *FOXC1* was downregulated in lt-NES^®^ cells following ZIKV infection, it could be a contributing factor to the development of congenital ZIKV syndrome.

FOXC1 further plays a role in oxidative stress response, whereby decreased FOXC1 transcript and protein levels were found in HTM cells exposed to oxidative stress [70]. Oxidative stress has been implicated in various neurodegenerative disorders [71] and several Flaviviridae viruses are known to trigger ROS increase and to interfere with antioxidant systems, which affects the virus life cycle and the cellular metabolism [72,73]. Hepatitis C virus (HCV) impairs the Nrf2 mediated antioxidant response, which in turn favors the release of viral particles. In HCV-positive cells, sMafs are bound to NS3 (nonstructural protein 3) and delocalized from the nucleus to the replicon surface, thereby trapping free Nrf2 and preventing its entry into the nucleus [31,32,72]. Impaired Nrf2/ARE-signaling contributes to elevated ROS levels, which in turn trigger the induction of autophagy, a pathway that is often manipulated by positive stranded RNA viruses to facilitate their replication and viral release. The crosstalk between oxidative stress and autophagy can support exosome-mediated release of viral particles, since autophagy and exocytosis both involve membrane trafficking and are functionally connected [72,74,75].

In this study, we found significantly higher levels of oxidative modified proteins after ZIKV infection, reflecting the induction of oxidative stress following ZIKV infection. Immunofluorescence analysis showed a colocalization of sMaf proteins with either ZIKV Uganda or ZIKV Polynesia envelope proteins and an accumulation of sMafs in perinuclear regions. It is possible that ZIKV, similar to HCV, interferes with sMafs to impair the induction of ARE-dependent genes, since major Nrf2 target genes including *HO-1*, *NQO1*, *GCL*, *GST* were not dysregulated upon ZIKV infection despite the induction of oxidative stress; however, further studies are needed to assess this [76,77,78]. Furthermore, in this study we investigated the colocalization of sMafs with the structural envelope proteins of ZIKV and not with non-structural proteins, whereas in the case of HCV the non-structural proteins NS3 interfere with sMafs. While the expression of these cytoprotective genes, which are crucial for the defense against oxidative stress, was not altered, the antioxidant gene Sestrin2 (*SESN2*) was significantly upregulated after infection. *SESN2* expression can be induced in response to ER stress by the transcription factors ATF4 (Activating Transcription Factor 4) or ATF6 (Activating Transcription Factor 6) and via the Nrf2/ARE pathway [79,80,81]. Consistent with our finding, Carr et al. recently described that *SESN2* was upregulated upon infection with Japanese encephalitis virus and ZIKV Uganda in neuroblastoma cells [82]. SESN2 is known to regulate ROS generation and to activate autophagy via inhibition of mTORC1, making it relevant as a therapeutic target and biomarker in various diseases [83]. Previous studies have shown that SESN2 levels are altered in patients with diseases including neurodegenerative disorders and plasma levels of SESN2 are positively correlated with the severity of those diseases [84,85,86]. It has been shown that suppression of miR-182-5p can lead to upregulation of *SESN2* in glioma and adenocarcinoma cell lines [53]. Interestingly, miR-182-5p was downregulated in lt-NES^®^ cells following ZIKV infection, which could be associated with the upregulation of *SESN2*. However, we acknowledge the limitation that further studies including gain and loss of function should be performed to further assess the interaction between miRNAs and mRNAs.

Collectively, our findings highlight multiple dysregulated host miRNAs and mRNAs upon ZIKV infection with roles in neurodevelopment and oxidative stress, which may contribute to a better understanding of the neurodegenerative phenotype induced by ZIKV infection. Furthermore, the lt-NES^®^ cells resulted as a suitable cell model to study dysregulation of miRNAs and genes by ZIKV infection and could be used for comparative studies of drug effects on neurodevelopment as well.

## Figures and Tables

**Table 1:** Comparative toxicogenomics database screening.

GeneSymbol	Fold Change ZIKV Uganda	*p*-Value ZIKV Uganda	Fold Change ZIKV Polynesia	*p*-Value ZIKV Polynesia
COL4A1	−2.1	0.0093	−1.2	0.635
MIR17HG	−2.3	0.0497	−1.3	0.749
TUBA1A	−2.2	2.56 × 10^−4^	−1.5	0.256
SLC2A1	−2.3	0.0239	1.0	0.806
ASNS	9.6	0.0314	2.9	0.006
STAMBP	2.5	1.07 × 10^−4^	1.4	0.975

Genes dysregulated by ZIKV Uganda > two-fold and by ZIKV Polynesia, which are associated with microcephaly according to the comparative toxicogenomics database [48].

**Table 2:** Top upregulated genes by ZIKV Uganda.

GeneSymbol	Fold Change ZIKV Uganda 24 hpi	Fold Change ZIKV Uganda 48 hpi	Fold Change ZIKV Uganda 72 hpi
OASL	1.6	49.9	766.7
CH25H	2.5	46.8	611.3
IFNB1	2.3	37.9	554.3
CXCL10	−1.3	40.8	521.3
IFIT2	1.0	30.7	218.8
IFIH1	2.1	38.1	176.4
PRDM16	8.1	23.5	141.9
CCL5	−1.3	10.3	117.1
CXCL11	1.1	12.8	104.1
GSC	1.2	9.0	60.4
BST2	1.8	26.6	59.0
IFI44	2.4	20.8	55.5
PKD1L2	2.8	9.6	49.9
DHX58	1.3	8.3	46.3
ATP4A	1.1	3.7	38.3
TMEM71	2.5	8.7	35.8
CEACAM1	1.4	6.8	35.8
NEURL3	1.2	10.5	34.0
IFI6	1.0	9.1	33.8

**Table 3:** Top upregulated genes by ZIKV Polynesia.

GeneSymbol	Fold Change ZIKV Polynesia 24 hpi	Fold Change ZIKV Polynesia 48 hpi	Fold Change ZIKV Polynesia 72 hpi
BST2	1.2	10.2	459.5
OAS1	−1.0	2.7	261.2
IFIH1	1.5	11.8	219.1
IFNB1	1.4	4.3	211.2
OASL	−1.1	6.3	193.1
CXCL10	1.0	4.2	147.1
IFITM1	1.4	4.8	133.1
HERC6	1.1	3.2	130.7
CH25H	1.5	3.0	126.7
IFI44	2.1	11.6	124.7
IFI6	−1.0	3.7	118.3
SAMD9L	−1.0	6.2	105.6
IFIT2	1.3	5.1	96.8
MX1	1.1	4.3	90.2
ISG15	−1.0	5.1	67.5
OAS2	−1.1	2.4	54.4
DDX60	1.0	4.3	52.0

**Table 4:** miR-4792: Diana mirPath v3 gne ontology analysis.

GO Category	*p*-Value	#Genes
Gene expression	2.49 × 10^−5^	12
Cellular nitrogen compound metabolic process	0.00013	37
Macromolecular complex assembly	0.00162	13
Protein complex assembly	0.01012	11
Cellular component assembly	0.01986	14
Biosynthetic process	0.02673	28
Biological process	0.02901	90
Negative regulation of oxidoreductase activity	0.03821	2
In utero embryonic development	0.03821	7

Biological processes, in which potential targets of miR-4792 are involved, which were predicted by TarBase.

**Table 5:** Differentially expressed genes (DEGs) during infection with ZIKV Polynesia at 72 hpi, which were annotated to GO-term ”response to oxidative stress”.

DEG	Fold Change ZIKV Polynesia
PNPT1	3.36
FOS	5.18
XBP1	2.06
PPARGC1A	2.06
KLF4	6.28
PML	3.76
TNFAIP3	4.96
STAT1	17.09
SLC7A11	2.11
JUN	2.10

**Table 6:** Differentially expressed genes (DEGs) during infection with ZIKV Polynesia at 72 hpi, which were annotated to GO-term ”response to endoplasmic reticulum stress”.

DEG	Fold Change ZIKV Polynesia
XBP1	2.06
EIF2AK2	4.72
ASNS	2.13
TRIB3	2.84
DDIT3	5.17
ATF3	5.56
HERPUD1	2.48
PPP1R15A	2.31
FBXO6	2.33
CHAC1	4.46

**Table 7:** EV associated miRNAs with decreased levels and their potential mRNA targets.

miRNA ID	Gene Targets (ID)
hsa-miR-26b-5p	TRIB3, FOS, HERPUD1, TNFAIP3, JUN, ASNS, CHAC1, ATF3
hsa-let-7g-5p	TRIB3, FBXO6, CHAC1
hsa-miR-17-5p	FOS, TNFAIP3, JUN, KLF4
hsa-miR-221-3p	FOS, PPP1R15A
hsa-miR-16-5p	HERPUD1, PML, EIF2AK2, JUN, ASNS, PPP1R15A, CHAC1, STAT1
hsa-miR-106b-5p	TNFAIP3
hsa-miR-374a-5p	TNFAIP3, EIF2AK2
hsa-miR-93-5p	TNFAIP3, JUN
hsa-miR-20a-5p	TNFAIP3
hsa-miR-186-5p	EIF2AK2, JUN, ASNS, CHAC1
hsa-miR-32-5p	JUN, KLF4
hsa-miR-218-5p	CHAC1
hsa-miR-340-5p	PPARGC1A

## Supplementary Materials

The following are available online at https://www.mdpi.com/1999-4915/12/11/1219/s1, Figure S1: Characterization of iLB-C-31f-r1 lt-NES^®^ cells, Figure S2: SNP analysis of iLB-C-31f-r1 lt-NES^®^ cells, Figure S3: Dysregulation of *ASNS* by ZIKV Uganda and Polynesia, Figure S4: Dysregulation of *BST2* by ZIKV Uganda and Polynesia, Figure S5: Dysregulation of miR-205 by ZIKV Uganda and Polynesia, Figure S6: Dysregulation of miR-4792 by ZIKV Uganda and Polynesia, Figure S7: Dysregulation of *FOXC1* by ZIKV Uganda and Polynesia, Figure S8: Dysregulation of *Sestrin2* and miR-182 by ZIKV Uganda and Polynesia, Figure S9: Gene ontology (GO) analysis of potential target genes of differentially expressed miRNAs, Table S1: Top downregulated genes by ZIKV Uganda at 72 hpi, Table S2: Downregulated genes by ZIKV Polynesia at 72 hpi, Table S3: Intracellular mature miRNAs dysregulated by ZIKV Polynesia and Uganda, Table S4: Intracellular mature miRNAs dysregulated by ZIKV Uganda, Table S5: EV-associated mature miRNAs dysregulated by ZIKV Polynesia, Table S6: EV-associated mature miRNAs dysregulated by ZIKV Uganda, Figure S10: Protein oxidation during ZIKV infection.
