# Supplementary material for: Impact of Zika Virus Infection on Human Neural Stem Cell MicroRNA Signatures
Source: Viruses. 2020 Oct 27;12(11):1219. doi: 10.3390/v12111219 (PMC7693339; doi:10.3390/v12111219)
Supplement: Supplementary file 1 [file viruses-12-01219-s001.pdf]

Supplemental Methods

Generation of lt-NES® cells

C31-r1 lt-NES® cells were established as described previously [1] with some modifications. Briefly, hiPSCs were detached with collagenase (Invitrogen, California, USA) and cultured in suspension on non-adhesive petri dishes to form aggregates in iPSC media without FGF2 but in the presence of 1 μM dorsomorphin and 5 μM SB431542. Medium was changed every second day, and at day 7 EBs were plated on tissue culture plates coated with poly-L-ornithine/laminin (both Sigma, Tokio, Japan). Neural rosette structures started to emerge about one week after plating and were carefully picked on day 14. Picked neural rosettes were cultured in suspension for two days in DMEM/F12, 2 mM L-glutamine, 20 mg/l additional insulin (Sigma, Tokio, Japan), 1.6 g/L glucose, 0.1 mg/ml penicillin/streptomycin, N2 supplement (1:100; Invitrogen, California, USA), then dissociated with trypsin, plated onto poly-L-ornithine/laminin-coated plates and propagated in the same medium but additionally supplemented with B27 (1 μL/mL, Invitrogen, California, USA), 10 ng/mL FGF2 and 10ng/ml EGF (both from R&D systems, Minnesota USA). Cells were passaged at a ratio of 1:2 – 1:3 every 2 – 3 days using trypsin.

Neural Differentiation of lt-NES® cells

Neural differentiation was induced by growth factor-withdrawal and cultivating the cells in Neurobasal Medium supplemented with B27 (1:50, Invitrogen, California, USA) and DMEM/F12 supplemented with N2 (1:100) mixed at a 1:1 ratio. 300 ng/ml cAMP was added to the differentiation medium. Cultures were analysed after 6 – 8 weeks.

Immunocytochemical analysis

Cells were fixed with 4% PFA in PBS for 10 min at room temperature and blocked in 10% FCS (Invitrogen, California, USA) in PBS. Cells were blocked in 0.1% Triton X-100 (Sigma, Tokio, Japan) and 10% FCS in PBS, incubated with the primary antibodies for 16 hours at 4°C, washed three times, incubated with secondary antibodies for 1 hour at room temperature, washed three times and counterstained with DAPI and mounted with Mowiol 4-88. Primary antibodies: PAX6 (1:300, Covance, New Jersey, USA), SOX1 (1:100, Millipore, Massachusetts, USA), SOX2 (1:500, R&D Systems, Minnesota, USA), Nestin (1:600, R&D Systems, Minnesota, USA), PLZF (1:50, Calbiochem, Massachusetts, USA), ZO-1 (1:100, Zymed, Massachusetts, USA), beta III-tubulin (1:2500, Covance, New Jersey, USA), MAP2ab (1:250, Chemicon, Limburg a. d. Lahn, Germany), GAD1/2 (1:1000, Chemicon, Limburg a. d. Lahn, Germany), NeuN (1:50, Chemicon, Limburg a. d. Lahn, Germany) and GABA (1:800, Sigma, Tokio, Japan). Secondary antibodies were Alexa488 anti-ms, Alexa555 anti-ms, Alexa488 anti-rb, Alexa555 anti-rb (all 1:1000, Invitrogen, California, USA), Cy3 anti-rat (1:300, Jackson/Dianova, Hamburg, Germany).

SNP analysis

SNP analyses were performed at the Institute of Human Genetics, University Hospital Bonn, Germany, using the PsychArray-24 v1.1. Data was analyzed using GenomeStudio (Illumina, California, USA).

1. Koch, P.; Opitz, T.; Steinbeck, J.A.; Ladewig, J.; Brustle, O. A rosette-type, self-renewing human ES cell-derived neural stem cell with potential for in vitro instruction and synaptic integration. *Proceedings of the National Academy of Sciences* **2009**, *106*, 3225–3230, doi:10.1073/pnas.0808387106.

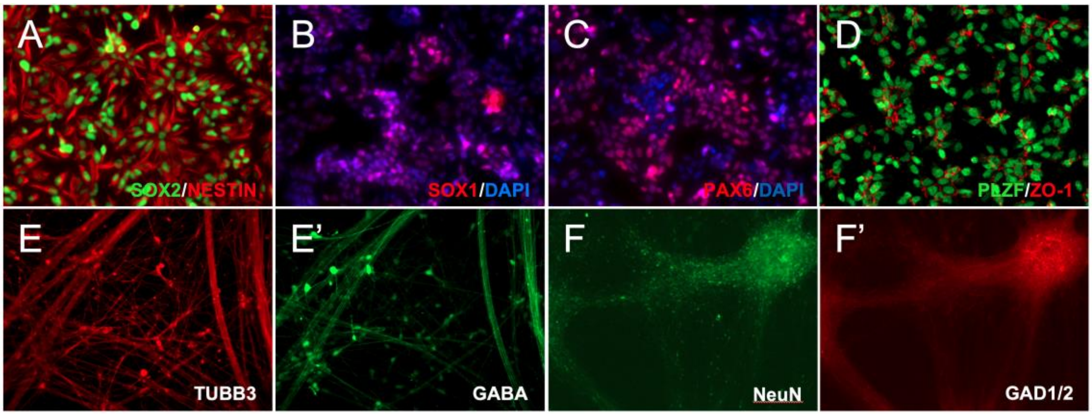

Figure S1. Characterization of iLB-C-31f-r1 lt-NES® cells. (**A-D**) iLB-C-31f-r1 lt-NES® cells express the neural stem cell markers SOX2, Nestin (**A**) and SOX1 (**B**), PAX6 (**C**) as well as the neural rosette marker PLZF (**D**). Expression of the tight junction protein ZO-1 (**D**) shows typical apical localization within neural rosette formations. (**E, F**) Upon growth factor withdrawal, iLB-C-31f-r1 lt-NES® cells give rise to neurons expressing TUBB3 (**E**) and the mature neuronal marker NeuN (**F**). Co-staining for the neurotransmitter GABA (**E′**) as well as glutamate decarboxylase (GAD1/2) (**F′**) reveals a dominant fraction of GABAergic inhibitory neurons in spontaneously differentiated cultures.

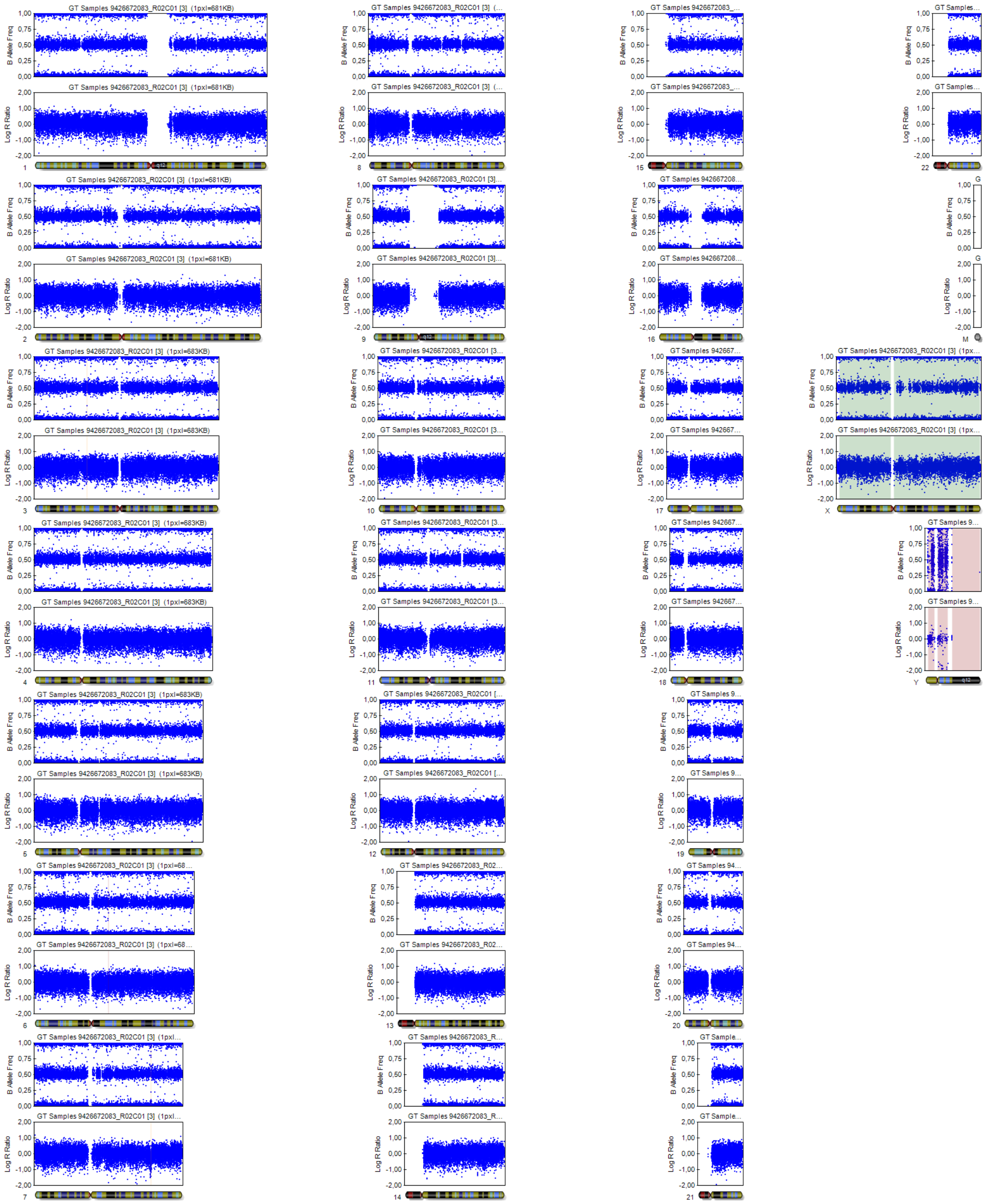

Figure S2. SNP analysis of iLB-C-31f-r1 lt-NES® cells. Whole-genome single nucleotide polymorphism (SNP) genotyping was performed after whole-genome amplification, DNA fragmentation and hybridization to bead-bound oligomers on PsychArray chip (Illumina, California, USA) at the Institute of Human Genetics (University of Bonn). Data were analyzed using Illumina GenomeStudio (Illumina, California, USA).

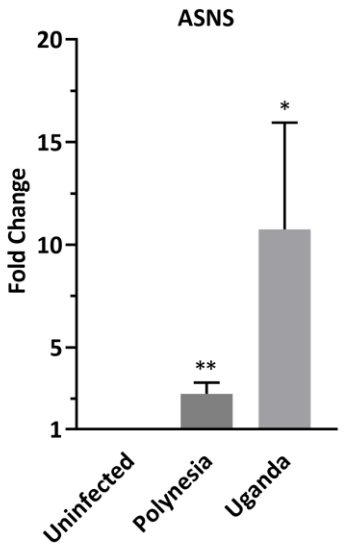

Figure S3. Dysregulation of *ASNS* by ZIKV Uganda and Polynesia. Relative *ASNS* mRNA levels measured by qRT-PCR presented as fold change compared to uninfected control cells at 72 hpi at MOI of 0.1. Error bar represents the mean ± SEM of n = 3 biological replicates; *p < 0.05; **p < 0.01.

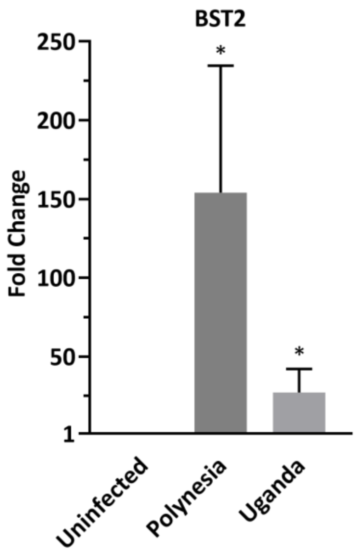

Figure S4. Dysregulation of *BST2* by ZIKV Uganda and Polynesia. Relative *BST2* mRNA levels measured by qRT-PCR presented as fold change compared to uninfected control cells at 72 hpi at MOI of 0.1. Error bar represents the mean ± SEM of n = 3 biological replicates; *p < 0.05.

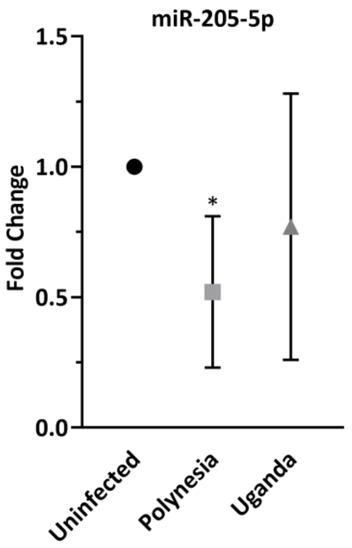

Figure S5. Dysregulation of miR-205 by ZIKV Uganda and Polynesia. Relative miR-205 levels measured by qRT-PCR presented as fold change compared to uninfected control cells at 48 hpi at a MOI of 0.1. Error bar represents the mean ± SEM of n = 3 biological replicates; *p < 0.05.

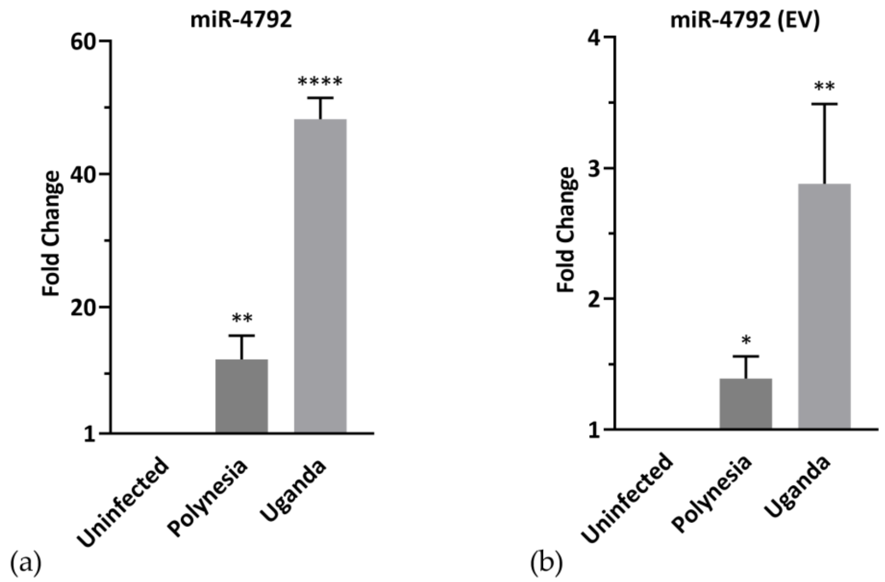

Figure S6. Dysregulation of miR-4792 by ZIKV Uganda and Polynesia. (**a**) Relative intracellular miR-4792 levels measured by qRT-PCR presented as fold change compared to uninfected control cells at 72 hpi at MOI of 0.1. Error bar represents the mean ± SEM of n = 3 biological replicates; **p < 0.01; ****p < 0.0001. (**b**) Relative EV-derived miR-4792 levels measured by qRT-PCR presented as fold change compared to uninfected control cells at 48 hpi at MOI of 1. Error bar represents the mean ± SEM of n = 3 biological replicates; *p < 0.05; **p < 0.01.

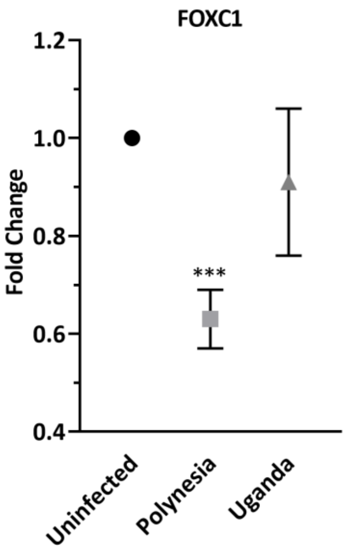

Figure S7. Dysregulation of *FOXC1* by ZIKV Uganda and Polynesia. Relative *FOXC1* mRNA levels measured by qRT-PCR presented as fold change compared to uninfected control cells at 72 hpi at MOI of 0.1. Error bar represents the mean ± SEM of n = 3 biological replicates; ***p < 0.001.

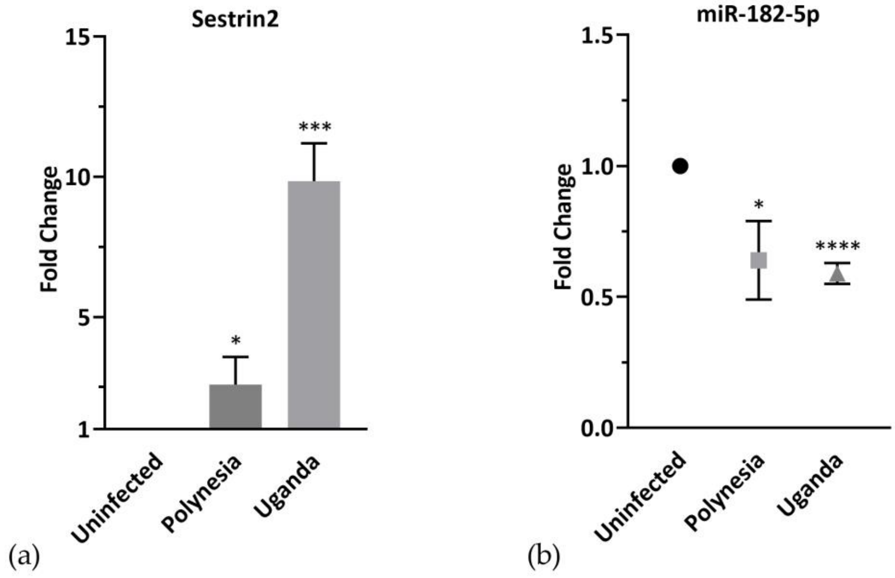

Figure S8. Dysregulation of *Sestrin2* and miR-182 by ZIKV Uganda and Polynesia. (**a**) Relative *Sestrin2* mRNA levels measured by qRT-PCR presented as fold change compared to uninfected control cells at 72 hpi at MOI of 0.1. Error bar represents the mean ± SEM of n = 3 biological replicates; *p < 0.05; ***p < 0.001. (**b**) Relative miR-182-5p levels measured by qRT-PCR presented as fold change compared to uninfected control cells at 72 hpi at MOI of 0.1. Error bar represents the mean ± SEM of n = 3 biological replicates; *p < 0.05; ****p < 0.0001.

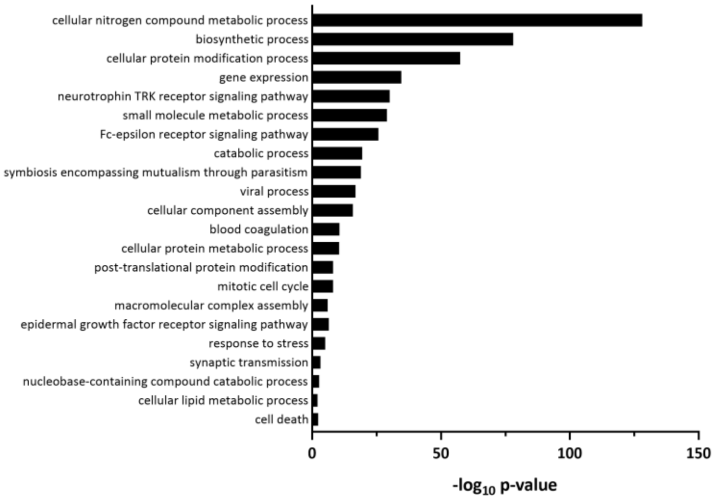

Figure S9. Gene ontology (GO) analysis of potential target genes of differentially expressed miRNAs. GO analysis of biological processes of potential target genes of miRNAs which were dysregulated only by ZIKV Uganda.

Table S1. Top downregulated genes by ZIKV Uganda at 72 hpi.

GeneSymbol	Fold Change ZIKV Uganda 72 hpi
LSP1	-7.4
COL9A1	-5.6
MIR670HG	-5.1
CTSC	-4.6
HES5	-4.4
CCDC177	-4.4
SMOC1	-4.2
HES3	-4.2
KCNS3	-4.0
RNASE4	-4.0
AGPS	-3.9
LIX1	-3.9
L3MBTL3	-3.8
GAL3ST3	-3.8
TRHDE-AS1	-3.8
OVOS2	-3.8
TPM3	-3.8
TARDBP	-3.7
LMO3	-3.7

Table S2. Downregulated genes by ZIKV Polynesia at 72 hpi.

GeneSymbol	Fold Change ZIKV Polynesia 72 hpi
YBX1	-2.6
PIAS1	-2.2
RAD1	-2.1
AGPAT9	-2.1
ADAMTS8	-2.0
LRP2	-2.0

Table S3. Intracellular mature miRNAs dysregulated by ZIKV Polynesia and Uganda.

miRNA ID<br>ZIKV Polynesia	log2 Fold Change<br>ZIKV Polynesia	log2 Fold Change<br>ZIKV Uganda
hsa-miR-6786-5p	9.20	11.32
hsa-miR-6090	8.58	10.18
hsa-miR-6746-3p	8.02	9.67
hsa-miR-5690	7.96	9.55
hsa-miR-6075	7.93	10.32
hsa-miR-718	7.78	9.55
hsa-miR-6084	7.55	9.75
hsa-miR-3195	7.08	9.20
hsa-miR-4667-5p	6.88	9.86
hsa-miR-6126	6.75	9.53
hsa-miR-3659	6.56	8.21
hsa-miR-4763-5p	6.48	8.94
hsa-miR-3621	6.47	8.68
hsa-miR-211-5p	6.46	9.56
hsa-miR-4634	6.37	8.46
hsa-miR-887-3p	6.36	7.92
hsa-miR-4800-5p	6.27	8.47
hsa-miR-638	6.23	9.04
hsa-miR-7161-3p	6.21	8.05
hsa-miR-4787-5p	6.18	8.09
hsa-miR-186-3p	6.07	7.36
hsa-miR-3614-3p	5.99	8.94
hsa-miR-4459	5.95	8.82
hsa-miR-6088	5.88	8.40
hsa-miR-6087	5.77	8.32
hsa-miR-4662a-5p	5.74	6.52
hsa-miR-3692-5p	5.70	7.67
hsa-miR-4449	5.69	7.33
hsa-miR-4516	5.57	8.12
hsa-miR-6509-5p	5.54	8.19
hsa-miR-6724-5p	5.46	8.30
hsa-miR-1181	5.41	7.39
hsa-miR-3960	5.37	7.62
hsa-miR-611	5.36	6.87
hsa-miR-3656	5.27	7.22
hsa-miR-1343-5p	5.25	8.61
hsa-miR-3141	5.24	7.15
hsa-miR-4792	5.14	7.60
hsa-miR-5787	5.07	7.16
hsa-miR-4743-5p	5.06	6.69
hsa-miR-6836-3p	5.01	7.30
hsa-miR-4654	4.98	7.41
hsa-miR-6510-5p	4.67	6.38
hsa-miR-6089	4.65	7.09
hsa-miR-759	4.47	6.47
hsa-miR-874-5p	4.21	7.92
hsa-miR-6836-5p	4.09	6.74
hsa-miR-6873-3p	4.08	6.19
hsa-miR-4800-3p	4.07	5.77
hsa-miR-1469	4.00	5.60
hsa-miR-6793-5p	3.93	6.67
hsa-miR-875-5p	3.86	6.57
hsa-miR-6876-3p	3.84	5.14
hsa-miR-4508	3.81	5.71
hsa-miR-6861-5p	3.78	6.99
hsa-miR-663a	3.78	5.99
hsa-miR-6870-5p	3.69	6.39
hsa-miR-6790-3p	3.56	5.91
hsa-miR-5585-5p	3.53	7.04
hsa-miR-4739	3.49	4.95
hsa-miR-4497	3.24	4.94
hsa-miR-183-5p	3.01	4.63
hsa-miR-7641	2.84	6.26
hsa-miR-4771	2.76	5.86
hsa-miR-589-5p	2.06	4.22
hsa-miR-92a-1-5p	1.88	3.12
hsa-miR-1261	1.20	3.59
hsa-miR-125b-1-3p	1.19	3.90
hsa-miR-7704	3.91	6.14
hsa-miR-182-5p	-1.51	-1.40

Table S4. Intracellular mature miRNAs dysregulated by ZIKV Uganda.

miRNA ID<br>ZIKV Polynesia	log2 Fold Change<br>ZIKV Uganda
hsa-miR-5701	6.15
hsa-miR-619-5p	3.48
hsa-miR-212-5p	2.82
hsa-miR-99b-3p	1.69
hsa-miR-99b-5p	1.35
hsa-let-7e-5p	1.28
hsa-miR-181a-2-3p	1.18
hsa-miR-125a-5p	1.09
hsa-miR-21-5p	1.00
hsa-miR-1180-3p	-1.13
hsa-miR-92b-3p	-1.16
hsa-miR-127-3p	-1.18
hsa-miR-493-5p	-1.21
hsa-miR-543	-1.21
hsa-miR-4652-3p	-1.26
hsa-miR-335-3p	-1.26
hsa-miR-654-3p	-1.27
hsa-miR-92a-3p	-1.30
hsa-miR-30c-5p	-1.33
hsa-miR-431-5p	-1.34
hsa-miR-28-5p	-1.40
hsa-miR-301a-5p	-1.44
hsa-miR-328-3p	-1.47
hsa-miR-1260b	-1.49
hsa-miR-4456	-1.56
hsa-miR-411-3p	-1.59
hsa-miR-484	-1.68
hsa-miR-151a-5p	-1.76
hsa-miR-4473	-2.15
hsa-miR-4455	-3.12

Table S5. EV-associated mature miRNAs dysregulated by ZIKV Polynesia.

miRNA ID ZIKV Polynesia	log2 Fold Change ZIKV Polynesia
hsa-miR-106b-5p	-2.66
hsa-miR-374a-5p	-2.58
hsa-miR-26b-5p	-2.49
hsa-miR-93-5p	-2.22
hsa-miR-32-5p	-2.04
hsa-miR-17-5p	-1.96
hsa-miR-301b	-1.91
hsa-miR-16-5p	-1.87
hsa-miR-20a-5p	-1.76
hsa-miR-103b	-1.76
hsa-miR-218-5p	-1.71
hsa-miR-186-5p	-1.52
hsa-miR-135a-5p	-1.47
hsa-miR-345-5p	-1.46
hsa-miR-221-3p	-1.37
hsa-miR-135b-5p	-1.30
hsa-let-7g-5p	-1.30
hsa-miR-340-5p	-1.12
hsa-miR-30a-3p	1.10
hsa-miR-4792	1.39
hsa-miR-423-5p	1.38
hsa-miR-99b-5p	1.42
hsa-miR-6131	1.43
hsa-miR-4488	1.51
hsa-miR-181a-2-3p	1.64

Table S6. EV-associated mature miRNAs dysregulated by ZIKV Uganda.

miRNA ID ZIKV Uganda	log2 Fold Change ZIKV Uganda
hsa-miR-5684	-2.77
hsa-miR-3907	-2.05
hsa-miR-8078	-1.93
hsa-miR-1273g-3p	-1.91
hsa-miR-4492	-1.84
hsa-miR-4508	-1.82
hsa-miR-6743-3p	-1.77
hsa-miR-149-3p	-1.71
hsa-miR-139-3p	-1.69
hsa-miR-6836-3p	-1.55
hsa-miR-663a	-1.52
hsa-miR-4800-3p	-1.46
hsa-miR-7704	-1.44
hsa-miR-1246	-1.43
hsa-miR-92a-1-5p	-1.38
hsa-miR-4488	-1.35
hsa-miR-8089	-1.33
hsa-miR-4785	-1.33
hsa-miR-1908-3p	-1.19
hsa-miR-619-5p	-1.18
hsa-miR-6724-5p	-1.06
hsa-miR-1260a	-1.03
hsa-miR-3960	-1.01
hsa-miR-20b-5p	1.05
hsa-miR-148a-3p	1.09
hsa-let-7f-5p	1.10
hsa-miR-103a-3p	1.10
hsa-miR-30a-5p	1.30
hsa-miR-16-5p	1.30
hsa-miR-106b-5p	1.54
hsa-miR-3529-3p	1.62
hsa-miR-7-5p	1.70
hsa-miR-374b-5p	1.70
hsa-miR-15b-5p	1.70
hsa-miR-95-3p	2.22
hsa-miR-374c-3p	2.23
hsa-miR-143-3p	2.95
hsa-miR-4792	2.88

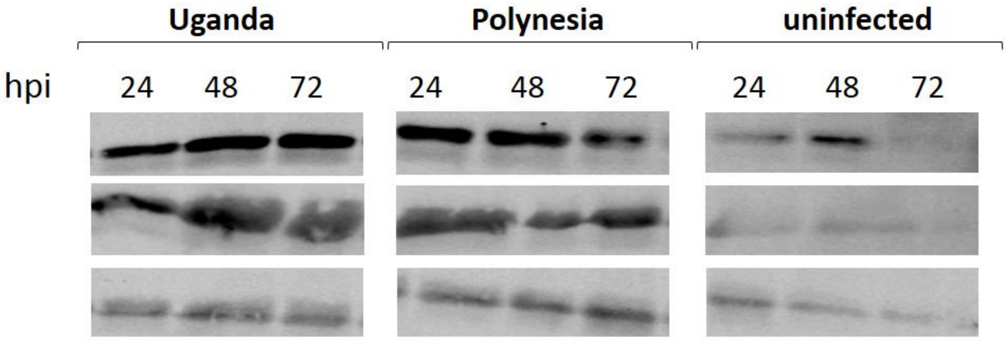

Figure S10. Protein oxidation during ZIKV infection. Oxyblot analysis of lysates derived from lt-NES® cells, which were infected with ZIKV Polynesia and Uganda at MOI of 1 and from uninfected lt-NES® cells. Protein oxidation was analyzed by incubation with 2,4,-dinitrophenylhydrazine (DNPH) for covalent modification of oxidative modified proteins.
